# Dynamically Rotating Magnetic Levitation to Characterize the Spatial Density Heterogeneity of Materials

**DOI:** 10.1002/advs.202300219

**Published:** 2023-05-01

**Authors:** Qiu‐Hua Gao, Peng‐Hui Song, Hong‐Xiang Zou, Zhi‐Yuan Wu, Lin‐Chuan Zhao, Wen‐Ming Zhang

**Affiliations:** ^1^ State Key Laboratory of Mechanical System and Vibration School of Mechanical Engineering Shanghai Jiao Tong University Shanghai 200240 P. R. China; ^2^ Hunan Provincial Key Laboratory of Vehicle Power and Transmission System Hunan Institute of Engineering 88 Fuxing East Road Xiangtan 411104 P. R. China; ^3^ SJTU Paris Elite Institute of Technology Shanghai Jiao Tong University Shanghai 200240 P. R. China

**Keywords:** dynamic rotation, equilibrium orientation, heterogeneity characterization, levitation stability, magnetic levitation, nonlinearity

## Abstract

Magnetic levitation (MagLev) is a promising technology for density‐based analysis and manipulation of nonmagnetic materials. One major limitation is that extant MagLev methods are based on the static balance of gravitational‐magnetic forces, thereby leading to an inability to resolve interior differences in density. Here a new strategy called “dynamically rotating MagLev” is proposed, which combines centrifugal force and nonlinear magnetic force to amplify the interior differences in density. The design of the nonlinear magnetic force in tandem with centrifugal force supports the regulation of stable equilibriums, enabling different homogeneous objects to reach distinguishable equilibrium orientations. Without reducing the magnetic susceptibility, the dynamically rotating MagLev system can lead to a relatively large change in orientation angle (∆*ψ* > 50°) for the heterogeneous parts with small inclusions (volume fraction VF = 2.08%). The rich equilibrium states of levitating objects invoke the concept of levitation stability, which is employed, for the first time, to characterize the spatial density heterogeneity of objects. Exploiting the tunable nonlinear levitation behaviors of objects provides a new paradigm for developing operationally simple, nondestructive density heterogeneity characterization methods. Such methods have tremendous potential in applications related to sorting, orienting, and assembling objects in three dimensions.

## Graphical abstract

Magnetic levitation(MagLev) is a promising technology for density‐based analysis and manipulation. In this study, a newstrategy called “dynamically rotating MagLev” is presented to characterize theintrinsic heterogeneity of objects through the interesting nonlinear levitationbehaviors. The dynamically rotating MagLev supports the regulation of stableequilibriums, enabling different homogeneous objects to reach distinguishableequilibrium orientations.

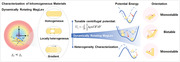

## 1 Introduction

Magnetic levitation (MagLev) is a cutting‐edge technology that offers a promising solution for density‐based analysis and manipulation of diamagnetic or weakly paramagnetic objects.^[^ ^1^, ^2^, ^3^, ^4^, ^5^ ^]^ Based on the balance of magnetic and buoyancy‐corrected gravitational forces, MagLev has been developed as a label‐free, magnetization‐removable physical technique for a wide range of materials, making it attractive for a variety of applications. The commonly used “standard” MagLev, containing a sample container sandwiched between two block permanent magnets with like poles facing each other, enables the density measurement (0.8‐3 g/cm^3^) of samples without knowing the mass and volume.^[^ ^1^ ^]^ More recently, a variety of MagLev configurations have been proposed to continuously improve the measurement performance. Compared to the “standard” MagLev, tilted^[^ ^6^ ^]^ and rotated^[^ ^7^ ^]^ MagLev configurations are designed to improve the measurement range and sensitivity, respectively. Using stacked magnet arrays, Ge et al.^[^ ^8^ ^]^ develops an integrated system that enables high‐throughput (a 96‐well plate) density measurement. New MagLev configurations using ring‐shaped magnets have also been developed, such as single‐ring MagLev,^[^ ^9^ ^]^ axial MagLev,^[^ ^10^ ^]^ axial‐circular MagLev,^[^ ^11^ ^]^ and ring‐shaped magnetic projection,^[^ ^12^ ^]^ providing a wide space for operation and observation. In addition to the permanent magnets mentioned above, single‐electromagnet levitation is also developed for adjustable density measurement.^[^ ^13^ ^]^ MagLev enables the density measurement of objects of arbitrary shape in various physical forms, such as small particles,^[^ ^6^, ^14^, ^15^ ^]^ crystal compounds,^[^ ^16^, ^17^ ^]^ thin polymeric films,^[^ ^18^ ^]^ soft gels,^[^ ^10^, ^19^ ^]^ and organic liquids.^[^ ^9^, ^20^ ^]^ The ability to resolve small differences in density makes it especially useful in material separation,^[^ ^7^, ^15^ ^]^ reaction monitoring,^[^ ^21^, ^22^, ^23^ ^]^ biological characterization of single cells,^[^ ^24^, ^25^, ^26^ ^]^ and disease diagnosis.^[^ ^27^, ^28^, ^29^ ^]^ Furthermore, MagLev can be utilized for quality control,^[^ ^30^, ^31^, ^32^, ^33^ ^]^ 3D assembly of both inanimate and living matter,^[^ ^34^, ^35^, ^36^ ^]^ noncontact orientation.^[^ ^37^, ^38^ ^]^ Despite the considerable advantages and rapid developments of MagLev or its variants, a major limitation lies in the fact that all density‐based analyses and manipulations are performed based on the balance of gravitational and magnetic forces. The constrained static principle leads to an inability to resolve the interior differences in density of the levitating objects, particularly those with gradient density. Therefore, there is a pressing need to develop a new paradigm of dynamic regulation that offers a higher degree of freedom for the characterization of inhomogeneous materials.

Rather than improving the measurement process by changing the magnetic force, our group has recently proposed a rotating‐mode MagLev method that enables flexible and convenient procedures to measure the average density of objects.^[^ ^39^, ^40^ ^]^ An approximately linear magnetic field is generated by four identical NdFeB magnets (every two magnets are arranged coaxially with like poles facing), making the average density measurement more straightforward. In this work, we further explore the MagLev combined with the centrifugal force to characterize the mass density of objects. Different from our previous works, we focus on characterizing the density distribution characteristics within the objects, and propose a new strategy to identify the density heterogeneity of materials by a series of levitation behaviors. The magnetic force generated by a pair of ring‐shaped magnets in tandem with the centrifugal force renders the levitation system kinetics inherently nonlinear and tunable, which allows different heterogenous objects to reach distinguishable equilibrium states. We investigate the effect of density heterogeneity and nonlinear magnetic field on the levitation position and orientation, and develop a mechanical model that quantitively describes the dynamic behavior of the inhomogeneous objects. In contrast to the static principle‐based “standard” MagLev in previous studies,^[^ ^1^, ^2^, ^11^, ^41^ ^]^ our work highlights the potential for characterizing the intrinsic heterogeneity of objects through the rich levitation behaviors offered by the introduced nonlinearity.

## 2 Design of Dynamically Rotating MagLev System

### 2.1 Working Principle

The general working principle of MagLev devices has been described in detail in previous works.^[^ ^1^, ^2^, ^41^ ^]^ Briefly, the “standard” MagLev configuration consists of two identical magnets with like‐poles facing each other and a sample container filled with paramagnetic solution sandwiched between the magnets (see Figure S1, Supporting Information for more details), forming a potential well at the midpoint. At equilibrium, the buoyancy‐corrected gravitational force (***F***_g_) acting on the object balances the magnetic force (***F***_mag_). The average density of the levitating object is proportional to the levitation height—the distance of the centroid of the sample to the top surface of the bottom magnet, and the distribution of density and its shape determines the levitation orientation. However, the static MagLev leads to an inability to resolve interior differences in density as the objects with different spatial distribution of density may exhibit the same equilibrium state.

**Figure 1:**
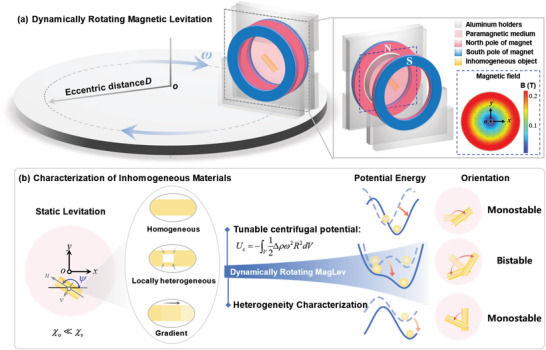
Dynamically rotating MagLev to characterize the spatial density heterogeneity of inhomogeneous materials. a) Schematic describing dynamically rotating MagLev system. The system mainly consists of the MagLev configuration that is eccentrically fixed on a spinning disc. The origin of the MagLev frame of reference is placed at the center of the middle plane of the two magnets with coordinates (*x*, *y*, *z*), and the origin of the body‐fixed, principal frame of reference is fixed at the centroid of the sample with coordinates (*u*, *v*, *w*). The orientation *ψ* is defined as the angle between the *x*‐ and *u*‐ axes. b) Working principle of the dynamically rotating MagLev. The rotating MagLev system supports the regulation of levitation stability by tuning the rotation parameters, enabling different inhomogeneous objects to exhibit distinguishable equilibrium orientations. The homogeneous objects are featured by a monostable rightward‐inclined orientation in the magnetic field. The locally heterogeneous objects exhibit an asymmetric bistable equilibrium orientation or bistable‐monostable orientation transitions, depending on the size of the interior heterogeneity. The objects with gradient density display a monostable leftward‐inclined orientation.

The point of departure of the dynamically rotating MagLev is to amplify the interior differences in density of the levitating objects by designing a centrifugal force. The proposed dynamically rotating MagLev system mainly consists of a MagLev configuration that is eccentrically fixed on a rotating disc, undergoing rotation relative to the direction of gravity (**Figure** **1**a). In order to characterize the spatial density heterogeneity, we need to obtain the quantitative relationship between the orientation angle and centrifugal force. As shown in Figure S2 (Supporting Information), only when the line formed by the shifted mass center and centroid is oriented in the middle plane can the inclined angle caused by the heterogeneity be observed. To address this issue, we choose to use a pair of ring‐shaped magnets with like poles facing each other and a central axis perpendicular to the gravity vector (Figure 1a). A transparent container filled with a paramagnetic solution is fixed between the magnets. We place the origin of the MagLev frame of reference at the center of the middle plane of the two magnets with coordinates (*x*, *y*, *z*) and the origin of the body‐fixed, principal frame of reference at the centroid of the sample with coordinates (*u*, *v*, *w*). Figure S3 (Supporting Information) plots the distribution of the magnetic field in the *x*‐*y* plane, *y*‐*z* plane, and *x*‐*z* plane. This distribution feature of the magnetic field controls the alignment of levitating objects in the *x*‐*y* plane as the orientation in the *x*‐*y* plane ensures the minimum energy. We define *ψ* as the orientation angle of the levitating object, i.e., the angle between the *x*‐ and *u*‐ axes.

Figure 1b illustrates the working principle of the dynamically rotating MagLev system. A nonconvex potential energy landscape is responsible for the existence of stable equilibrium states (including the levitation position and angular orientation). The gravitational potential energy, magnetic potential energy, and centrifugal potential energy collectively contribute to the effective potential energy of levitating objects. Thereinto, the magnetic potential energy generated by the ring‐shaped magnets renders the system kinetics inherently nonlinear, which is responsible for the existence of nonlinear levitation behaviors. The design of centrifugal potential energy enables the rich and highly tunable equilibrium states of the levitating objects, thus allowing to explore the interplay between the equilibrium orientation and density heterogeneity in multiple dimensions. Here we divide the interior density distribution of objects into three categories: homogeneous, locally heterogeneous, and gradient. The interior heterogeneity of objects generally can be equated to one of these three types mentioned above. The effective energy of homogeneous object is characterized by one potential well, and the position where the effective potential energy is minimized can be tuned by varying the centrifugal potential energy. Then the homogeneous object is featured by a monostable rightward‐inclined orientation in the magnetic field. For the statically levitated objects with local heterogeneity in density, symmetric angular orientations generally occur as the effective energy potential has two wells of equal height. When the MagLev system is in rotation, the locally heterogeneous object deviates from the magnetic field center in the rotational rigid‐body mode, exhibiting an asymmetric bistable equilibrium oblique orientation. When the centrifugal force is further increased, a transition from the asymmetric bistable to monostable orientation occurs, which is unique to the locally heterogeneous objects. Gradients in density are commonly found in functionally graded structures and materials.^[^ ^42^ ^]^ In the MagLev devices based on the static principle, objects with different gradients in density show an indistinguishable vertical orientation (*ψ* = *π*/2), with the less dense side facing upward. By imposing the centrifugal force, the differences in density gradient can be characterized by the monostable leftward‐inclined orientation angles. Therefore, the proposed dynamically rotating MagLev, with superior maneuverability and tunability, can characterize the spatial density heterogeneity of materials by a series of levitation behaviors.

### 2.2 Modeling of Dynamically Rotating MagLev System

According to the Coulombian approach,^[^ ^43^, ^44^ ^]^ the axially magnetized magnet ring is represented by two annular planes which correspond to the upper and lower faces. The upper one is charged with a surface magnetic pole density +σ* and the lower one is charged with the opposite surface magnetic pole density −σ*. Then the total magnetic field created by the ring can be obtained by linearly superimposing the magnetic fields generated by the upper and lower faces. The magnetic field ***H*** (*r*, *z*) created by the ring magnet at any point *M*(*r*, *z*) can be given by

(1) $$\begin{array}{c} H\left(r,z\right)=\frac{\sigma^{*}}{4\pi \mu_{0}}\int_{\theta =0}^{\theta =2\pi }\int_{r_{1}=r_{\mathrm{in}}}^{r_{1}=r_{\mathrm{out}}}\left(\frac{P_{1+}M}{{\left|P_{1+}M\right|}^{3}}r_{1}-\frac{P_{1-}M}{{\left|P_{1-}M\right|}^{3}}r_{1}\right)dr_{1}d\theta \end{array}$$

where *r*_out_ and *r*_in_ denote the outer and inner radius of the magnet, *P*_1+_ is a point on the upper face, and *P*_1−_ is a point on the lower face. Integration of Equation 1, we can obtain the magnetic field components *H_r_* (*r*, *z*), *H_*θ*_* (*r*, *z*), and *H_z_* (*r*, *z*) in the cylindrical coordinates. The analytical calculation of the magnetic field is detailed in the Supplementary Information. Based on the coordinate system transformation and vector superposition, we can obtain the magnetic induction intensity ***B*** (*x*, *y*) of the proposed configuration, which can be expressed as follows:

(2) $$\begin{array}{c} B_{x}\left(x,y\right)=H_{r}\left(r,z\right)\cdot \frac{x}{\sqrt{x^{2}+y^{2}}}\\ B_{y}\left(x,y\right)=H_{r}\left(r,z\right)\cdot \frac{y}{\sqrt{x^{2}+y^{2}}} \end{array}$$

**Figure 2:**
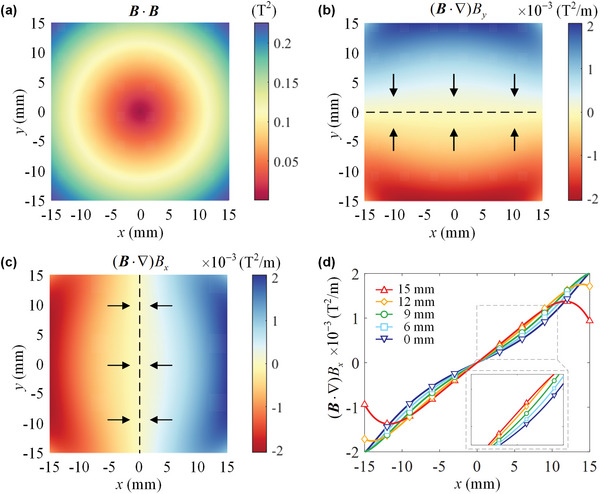
Characteristics of the magnetic field in the *x*‐*y* plane. a) The spatial profile of ***B*** · ***B*** in the *x*‐*y* plane, characterizing the magnitude of the magnetic potential energy. Panels show the distribution of b)(***B*** · ∇)*B_x_* and c) (***B*** · ∇)*B_y_* for varying *x* and *y*. Black arrows denote the direction of the magnetic force components ***F***_mag_ *_x_*, ***F***_mag_ *_y_*. d) Comparison of the relationship between (***B*** · ∇)*B_x_* and the position along the *x*‐axis with varying *y* (from 0 to 15 mm). The nonlinear effect becomes stronger when *y* increases.

Two ring‐shaped NdFeB magnets with the same dimension (*r*_out_ × *r*_in_ × *h* = 50 mm × 25 mm × 15 mm, N40) are positioned apart by *d* = 17 mm, the illustration of the geometric dimension is shown in Figure S4 (Supporting Information). The MagLev configuration results in a circularly symmetric magnetic field in each cross‐sectional plane, with the smallest magnetic induction intensity in the *x*‐*y* plane. The orientation can be aligned in the *x*‐*y* plane, and the equilibrium levitation can be characterized by its translation and rotational motion in the *x*‐*y* plane. **Figure** **2**a plots the spatial profile of ***B*** · ***B*** in the *x*‐*y* plane, depicting the magnitude distribution of magnetic potential energy. Figure 2b,c plot the spatial profile of the (***B*** · ∇)*B_y_* and (***B*** · ∇)*B_x_* in the *x*‐*y* plane, which also show the magnitude distribution of the nonlinear magnetic force ***F***_mag_ *_x_* and ***F***_mag_ *_y_*. To further quantitively characterize the nonlinearity of the magnetic force, we compare the relationship between (***B*** · ∇)*B_x_* and the position along the *x*‐axis with varying *y* (from 0 to 15 mm) in Figure 2d. The nonlinear relationship between (***B*** · ∇)*B_x_* and *x* is stronger when *y* increases. Thus, it's essential to consider the nonlinear effect of the magnetic field on the levitation orientation.

To predict the levitation behavior of the inhomogeneous objects, we develop a mechanical model to describe the effect of the magnetic field and spatial heterogeneity in density on the equilibrium position and orientation. For an inhomogeneous object levitated in the rotating MagLev system, we decompose its effective potential energy into gravitational, magnetic, and centrifugal contribution in the non‐inertial frame of references, that is,

(3) $$\begin{array}{ccc} U & = & U_{\mathrm{mag}}+U_{\mathrm{grav}}+U_{c}\\ & = & -\int_{V}\left(\frac{\Delta \chi }{2\mu_{0}}B^{2}+\Delta \rho \mathrm{gy}+\frac{1}{2}\Delta \rho \omega^{2}{\left(D+x\right)}^{2}\right)dV \end{array}$$

where *U*_mag_, *U*_grav_, and *U*_c_ represent the magnetic, gravitational, and centrifugal contributions to the effective potential energy, respectively. *V* is the volume of the object. *µ*_0_ = 4*π* × 10^−7^(*N*/A^2^) is the magnetic permeability of free space, and ***g*** is the acceleration due to gravity. ▵*χ* = *χ*_o_ − *χ*_s_ is the magnetic susceptibility of the object *χ*_o_ relative to the solution *χ*_s_, and ▵*ρ* = *ρ*_o_ − *ρ*_s_ is the density of the object *ρ*_o_ relative to the solution *ρ*_s_. The calculation of the magnetic susceptibility of the paramagnetic solution is detailed in the Supplementary Information. *x* and *y* represent the centroid position of the object in the magnetic field. ***B*** (*x*, *y*) represents the magnetic field vector in the *x*‐*y* plane. *ω* is the rotating speed of the system, and *D* is the distance from the center of the MagLev to the center of rotation. The object of arbitrary shape is heterogeneous, which is reflected as a function describing how density within objects are distributed in the body‐fixed frame of reference.

Stable equilibrium occurs where the local minimum of the effective potential energy *U* exists. Finding the equilibrium levitation involves minimizing simultaneously the energy associated with the levitation position as well as orientation, which can be given by

(4) $$\nabla U=0\Leftrightarrow \begin{array}{c} \frac{\partial U}{\partial x_{0}}=\frac{\partial U_{\mathrm{mag}}}{\partial x_{0}}+\frac{\partial U_{\mathrm{grav}}}{\partial x_{0}}+\frac{\partial U_{c}}{\partial x_{0}}=0\\ \frac{\partial U}{\partial y_{0}}=\frac{\partial U_{\mathrm{mag}}}{\partial y_{0}}+\frac{\partial U_{\mathrm{grav}}}{\partial y_{0}}+\frac{\partial U_{c}}{\partial y_{0}}=0\\ \frac{\partial U}{\partial \psi }=\frac{\partial U_{\mathrm{mag}}}{\partial \psi }+\frac{\partial U_{\mathrm{grav}}}{\partial \psi }+\frac{\partial U_{c}}{\partial \psi }=0 \end{array}$$

In this equation, we assume that the anisotropy of magnetic susceptibility has a negligible effect on the levitation since the magnetic susceptibility of the nonmagnetic object is much smaller than that of the paramagnetic solution. Then the object is considered to be homogeneous in magnetic susceptibility relative to the paramagnetic solution. For the nonlinear magnetic field ***B*** (*x*, *y*), a cubic polynomial is chosen to approximate the distribution of the magnetic field obtained from the Coulombian approach. The comparison of the magnetic field between the theoretical calculation and fitted results is shown in Figure S5 (Supporting Information). Numerically solving the resulting set of the multivariable Equation 4 provides the levitation position and orientation information in the magnetic field. The first and second equations in Equation 4 describe the gravitational, magnetic, and centrifugal forces balance at the equilibrium position, and the third equation indicates the gravitational, magnetic, and centrifugal torques balance at the equilibrium orientation. The equilibrium levitation state (*x*_0_, *y*_0_, *ψ*) will occur at the local minima of *U*, where ∇*U* = 0 and ∇^2^ *U* > 0.

## 3 Orientation of Homogeneous Objects in Dynamically Rotating MagLev

To demonstrate the effectiveness of the dynamically rotating MagLev system, we commenced by investigating the impact of nonlinear magnetic field on the levitation orientation using a homogeneous object. To reduce the influence of the geometry of the object on the levitation, we designed and fabricated a series of cylindrical rods, with a diameter of *d*_1_ = 6 mm and a length of *l*_1_ = 12 mm, by 3D printing (Stratasys, J750). The *u*‐axis is set to be aligned with the length axis of the rod. We define the equilibrium orientation as the angle *ψ* between the *u*‐ and *x*‐ axes and the equilibrium position as the distance *r* from the centroid of the rod to the center of the magnetic field. Before observing the orientation of the rod, we first measure the density of the printed material using the dynamically rotating MagLev. The density of a sphere made of the same white resin (Verowhite, Stratasys) is measured to be *ρ*_1_ = 1.203 g/cm^3^ by the previous rotating‐mode method (see Figure S8, Supporting Information).

**Figure 3:**
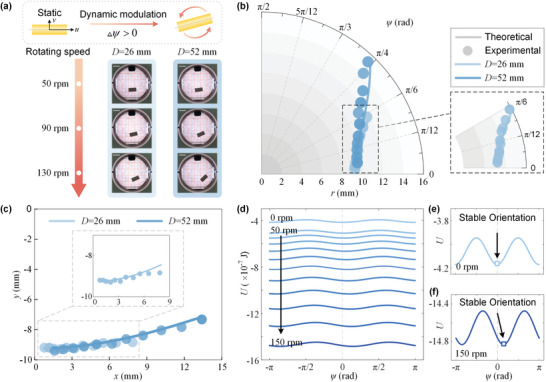
Orientations of the homogeneous rod (*d*_1_ = 6 mm, *l*_1_ = 12 mm) in the dynamically rotating MagLev. a) Illustration of the equilibrium orientation represented by the levitation angle *ψ*. Representative images of the homogeneous rod in the MagLev configuration when the centrifugal force is imposed. Scale bar, 10 mm. b) Evolution of levitation position and orientation for the varying rotating speeds (from 50 to 150 r min^−1^) and eccentric distances (*D* = 26 mm, 52 mm). c) Comparison between the theoretical results and experimental data of the equilibrium position as a function of the rotating speed. d) Quantitative characterization of potential energy landscape as a function of orientation angle *ψ* at different rotating speeds (from 50 to 150 r min^−1^). Comparison of the effective potential energy with respect to the orientation angle *ψ* at the rotating speeds of e) *ω* = 0 r min^−1^, and f) *ω* = 150 r min^−1^.

The homogenous rod is levitated in 1.78 m MnCl_2_ aqueous solution in the MagLev. The statically levitated homogeneous rod orients at *ψ* = 0 due to the balance of gravitational and magnetic force, an observation consistent with the previous report.^[^ ^37^ ^]^ In order to analyze the effect of centrifugal force on the levitation orientation, we carry out experiments at different rotating speeds (from 50 to 150 r min^−1^ in the increment of 10 r min^−1^) and eccentric distances (*D* = 26 mm, 52 mm). We capture the images of the levitating rod at different rotating speeds and measure the position as well as orientation angle (**Figure** **3**a).

In Figure 3b, we report the evolution of the equilibrium position *r* and orientation angle *ψ* for different rotating speeds and eccentric distances. It is energetically favorable and flexible for the rod to switch multiple equilibrium states by tuning the centrifugal potential energy. Both the theoretical and experimental results demonstrate that the homogeneous rod is featured by a rightward‐inclined orientation. As the centrifugal force increases (including the rotating speed *ω* and eccentric distance *D*), both equilibrium position and orientation increase. The centroid position of the homogeneous rod for varying rotating speed is shown in Figure 3c. The theoretical calculations match the experimental results, demonstrating that the mechanical model can effectively predict the equilibrium state of the homogeneous rod. Furthermore, Figure 3d shows a series of potential energy landscapes of the homogenous rod at different rotating speeds when the eccentric distance is set 26 mm. As the rotating speed increases, the effective potential energy decreases. For the static levitation of the homogeneous rod, i.e., the rotating speed of *ω* = 0 r min^−1^, the potential energy minimum occurs at *ψ* = 0 (as shown in Figure 3e). For the rotating speed of *ω* = 150 r min^−1^, as shown in Figure 3f, the unique potential minimum occurs at *ψ* = 0.497 rad, and the other values of *ψ* that account for energy extrema are not stable.

## 4 Characterization of Objects with Local Heterogeneity in Density

In order to construct local heterogeneity in density and enable flexible adjustment of position and size, we utilized the multi‐material 3D printer (Stratasys, J750) capable of printing materials of dissimilar densities. The symmetric cylindrical roads using white resin material (Verowhite, Stratasys) incorporated a series of inclusions made of transparent soft rubber material (Agilus 30, Stratasys). The schematic of the dimensions and body‐fixed frame of references of the rods are shown in Figure S9 (Supporting Information). The densities of these two printed materials are determined in the dynamically rotating MagLev (see Figure S8, Supporting Information), where the density of the white resin is *ρ*_1_ = 1.203 g cm^−3^ and the density of the soft rubber is *ρ*_2_ = 1.146 g cm^−3^.

**Figure 4:**
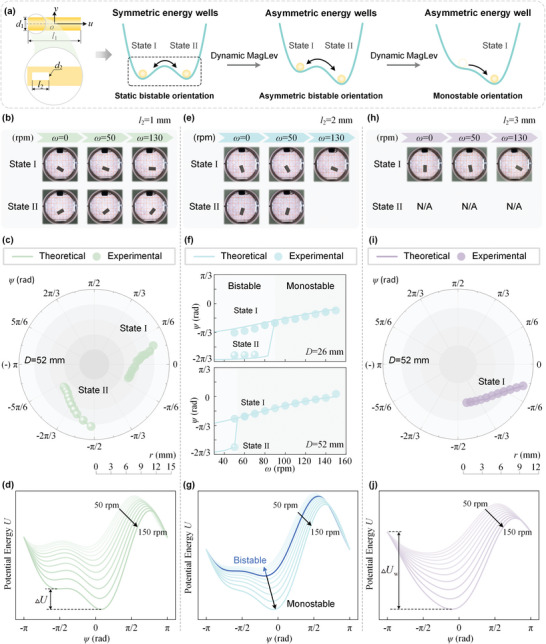
Characterization of objects with the local heterogeneity in density. a) Schematic of the evolution of the potential energy. The statically levitated object with a local heterogeneity shows a bistable orientation with symmetric potential energy wells. The potential energy landscape can be tuned by introducing the centrifugal potential to exhibit asymmetric wells, enabling bistable‐monostable orientation. b–d) Levitation states for the rod with the local heterogeneity defined by *l*_2_ = 1 mm: b) experimental images of the bistable orientation, c) equilibrium orientation and position, d) evolution of the effective potential energy as a function of rotating speed. e–g) Levitation states for the rod with the local heterogeneity defined by *l*_2_ = 2 mm: e) experimental images of the bistable‐monostable transition, f) plot of orientation angle *ψ* as a function of rotating speed *ω* for different eccentric distance, g) evolution of the effective potential energy from asymmetric potential wells to monostable potential well. h–j) Levitation states for the rod with the local heterogeneity defined by *l*_2_ = 3 mm: h) experimental images of monostable levitation, i) equilibrium orientation and position, j) comparison of the monostable potential well as a function of rotating speed.

Moreover, both theoretical and experimental investigations are conducted to analyze the dynamic responses of the rods containing inclusion cylinders of varying volumes, hence to elucidate the effect of local heterogeneity in density on the levitation orientation. Specifically, we focus on three representative kinds of rods (*l*_1_ = 12 mm, *d*_1_ = 6 mm) containing the cylindrical inclusions with different heights (*l*_2_ = 1, 2, and 3 mm), but with the same diameter of *d*_2_ = 3 mm and same inclusion distance (*t* = 2 mm) to the end face. The rods are levitated in 1.78 m MnCl_2_ aqueous solution in the MagLev. When statically levitated, the rods with the inclusion heights of *l*_2_ = 1 mm, 2 mm are in symmetric bistable orientation, while the rod with the inclusion height of *l*_2_ = 3 mm adopts a vertical orientation (see Figure S10, Supporting Information). In **Figure** **4**a, by imposing the tunable centrifugal potential energy, we introduce an asymmetry into the system, making the two energy minima asymmetric and different. Then the levitating rod features a bistable asymmetric orientation. When further decreasing the centrifugal potential energy, the energy barrier between the two potential wells may decrease. Thus, the levitation orientation transitions from bistable to monostable.

Figure 4b shows the representative snapshots of the bistable orientation of the rod at different rotating speeds. We report the comparison results predicted by theoretical model and performed by experiments for the rod with *l*_2_ = 1 mm in Figure 4c. The introduction of the centrifugal force enables the rod to levitate in an asymmetric bistable orientation. The orientation angle in the two states monotonically varies as the rotating speed increases, and the theoretical model can reliably predict the levitation orientation. A comparison of the centroid position of the levitating rod in the two states is plotted in Figure S11 (Supporting Information). Theoretical calculation and experimental results show that the centroid positions in State I and II are close but not the same since the local heterogeneity makes the centroid not coincide with the center‐of‐mass. Figure 4d plots the evolution of the effective potential energy as a function of the orientation angle *ψ*. For the rod with *l*_2_ = 1 mm, there exist two potential wells in the rotating speed range of 50–150 r min^−1^. As the rotating speed increases, the energy difference ▵*U*between the two potential wells increases, thus leading to an asymmetric bistable orientation.

Figure 4e shows the images of the levitating rod with *l*_2_ = 2 mm, visually demonstrating the transition from bistable to monostable. In Figure 4f, we report the evolution of the orientation angle *ψ* as a function of the rotating speed for two different eccentric distances (*D* = 26 mm and *D* = 52 mm). The dynamic response of the levitating rod for the eccentric distance of D = 26 mm reveals that the orientation is bistable when *ω* ≤ 90 r min−1, whereas the orientation is monostable when *ω*>90 r min−1. By contrast, the dynamic response for the eccentric distance of *D* = 52 mm reveals that the bistable orientation occurs within *ω* ≤ 50 r min^−1^. As shown in Figure 4g, we report the evolution of the potential energy *U* as a function of the orientation angle *ψ* for different rotating speeds. We find that the rotating speed plays an important role, as it directly affects the energy barrier between the neighboring energy wells. Since the relative energy barrier monotonically decreases as the rotating speed increases, the levitation orientation can be manipulated by tuning the rotating speed to make the energy barrier lower than one of the potential energy wells. Furthermore, the size of the local heterogeneity can be characterized by the centrifugal force for orientation transition. Typically, the larger the size of the local heterogeneity, the smaller the critical centrifugal force is required.

Finally, Figure 4h shows the representative images of the levitating rod at different rotating speed, demonstrating the monotonic variation of the orientation angle. In Figure 4i we report the evolution of the levitation orientation as a function of levitation position for the rod with *l*_2_ = 3 mm. The results indicate that the levitation state of the rod with *l*_2_ = 3 mm is monostable for any choice of the rotating speed, with the orientation angle *ψ*>‐*π*/2. The good agreement between the experimental and theoretical data verifies the predictive ability of our model. We then characterize the dynamic response by plotting the energy landscape as a function of orientation angle *ψ* for different rotating speeds. As the rotating speed increases, the effective potential well becomes deeper. Different from the results mentioned above, the total energy landscape of the rod only has one minimum, of which the occurrence varies unidirectionally with the rotating speed.

## 5 Comparison of Objects with Gradient in Density

**Figure 5:**
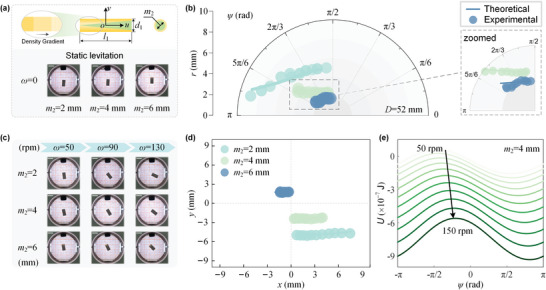
Characterization of objects with gradient in density. a) Images of the statically levitated rods with different gradients in density. All of the rods orient vertically. Scale bar, 10 mm. b) Evolution of the levitation orientation versus position compared by the theoretical results and experimental data. c) Images of the levitating rods at the rotating speeds of *ω* = 50, 90, and 130 r min^−1^. Scale bars, 10 mm. d) Plot of the centroid position in the magnetic field. e) Effective potential energy landscape as a function of the orientation angle *ψ* for the rod with *m*_2_ = 4 mm. The rotating speed is set from 50 to 150 r min^−1^.

It has been demonstrated that the objects with symmetric defects cannot be detected by the standard MagLev device due to no deviation from the centroid to the center‐of mass.^[^ ^30^ ^]^ Here we explore the application of dynamically rotating MagLev in density gradient characterization. Specifically, we consider the rods with different density gradient along the long axis. The 3D‐printed cone‐shaped inclusions with different end face diameters of *m*_2_ (*m*_2_ = 2, 4, and 6 mm) were made to construct a continuously systematic gradient. When statically levitated in 1.78 m MnCl_2_ aqueous solution in the MagLev configuration, the rods are all oriented vertically with the same angle (*ψ* = *π*/2) (**Figure** **5**a). In Figure 5b, we report the evolution of the levitation orientation versus position for different rotating speeds ranging from 50 to 150 r min^−1^. The rods with the gradient density exhibit a distinguishable leftward‐inclined monostable levitation orientation when the centrifugal force is imposed. Thereinto, the rod with the smallest gradient (*m*_2_ = 2 mm) adopts the largest orientation, with an angle of *ψ* = 2.91 rad when *ω* = 150 r min^−1^. Meanwhile, we find that the introduction of the centrifugal modulation is beneficial for amplifying the differences in gradient density as the difference in the orientation angle becomes larger as the rotating speed increases. Figure 5c shows the representative snapshots of the gradient levitating rods at different rotating speeds. With the centrifugal modulation, we can directly characterize the inherent density gradient by the equilibrium orientation and position. Theoretical results and experimental data indicate that the orientation angle monotonically increases as the rotating speed increases, with the centroid away from the center of the magnetic field (Figure 5d). In Figure 5e, we report the evolution of the potential energy landscape *U* as a function of the orientation angle *ψ* for the rod with the inclusion of *m*_2_ = 4 mm. It can be seen that the effective potential energy, with one tunable potential well, decreases as the rotating speed increases. Therefore, the rods with the gradient in density can be characterized by a series of leftward‐inclined orientations, and the differences in gradient can be characterized by the orientation angles.

In summary, both theoretical and experimental results have been conducted to demonstrate the effectiveness of the dynamically rotating MagLev configuration. The design of nonlinear magnetic force in tandem with centrifugal force enables an identification of the density characteristics (such as homogeneous, locally heterogeneous, and gradient density) through the rich levitation behaviors. Based on the new strategy, future work would focus on a data‐driven approach using multiple unique equilibrium states to realize defect detection and reconstruction of density distribution. Compared to existing MagLev configurations, however, there are also some constraints in terms of portability and miniaturization. Future work would also involve an optimization of the present device by designing a small‐scale, compact MagLev configuration and constructing an integrated driving‐controlling rotation system.

## 6 Conclusion

In this study, we present a novel strategy termed as “dynamically rotating MagLev” for characterizing the spatial density heterogeneity of materials. Different from extant MagLev configurations, the nonlinear magnetic field and tunable centrifugal force render the system kinetics inherently nonlinear, allowing different inhomogeneous objects to display distinguishable equilibrium orientations. We have investigated the effect of the nonlinear magnetic field and interior heterogeneity in density on the equilibrium orientation and position, and developed a mechanical model to describe the dynamic behavior of the levitating objects. Our results indicate that homogeneous objects exhibit a monostable rightward‐inclined orientation, while objects with local heterogeneity display an asymmetric bistable orientation, which undergoes a bistable‐monostable orientation transition upon increasing the centrifugal force. Without reducing the magnetic susceptibility of the medium or compromising the levitation stability, the dynamically rotating MagLev can lead to a relatively large change in orientation angle (∆*ψ* > 50°) for the heterogeneous parts with small inclusions (volume fraction VF = 2.08%). For the objects with different density gradients that cannot be detected in the “standard” MagLev, the differences in gradient can be characterized by the rightward‐inclined orientation angles. The dynamically rotating MagLev here serves as a representative example of employing multidimensional nonlinear dynamic levitation behavior for nondestructive characterization of materials. The ability to simultaneously characterize the spatial heterogeneity of inhomogeneous materials and manipulate the orientation of levitating objects of the proposed strategy suggests a wide range of opportunities for quality control of materials, sorting, and assembling objects in 3D.

## Conflict of Interest

The authors declare no conflict of interest.

## Supporting information

Supporting Information

## Data Availability

The data that support the findings of this study are available from the corresponding author upon reasonable request.
