# Supplementary material for: Dynamically Rotating Magnetic Levitation to Characterize the Spatial Density Heterogeneity of Materials
Source: Adv Sci (Weinh). 2023 May 1;10(20):2300219. doi: 10.1002/advs.202300219 (PMC10369266; doi:10.1002/advs.202300219)
Supplement: Supplementary file 1 — Supporting Information [file ADVS-10-2300219-s001.pdf]

ADVANCED SCIENCE
Open Access

Supporting Information

for *Adv. Sci.*, DOI 10.1002/advs.202300219

Dynamically Rotating Magnetic Levitation to Characterize the Spatial Density Heterogeneity of Materials

*Qiu-Hua Gao, Peng-Hui Song, Hong-Xiang Zou, Zhi-Yuan Wu, Lin-Chuan Zhao and Wen-Ming Zhang**

Supplementary Information for

Dynamically Rotating Magnetic Levitation to Characterize the Spatial Density Heterogeneity of Materials

Qiu-Hua Gao[1], Peng-Hui Song[1], Hong-Xiang Zou[2], Zhi-Yuan Wu[1], Lin-Chuan Zhao[1], Wen-Ming Zhang[1, 3*]

[1] State Key Laboratory of Mechanical System and Vibration, School of Mechanical Engineering, Shanghai Jiao Tong University, Shanghai 200240, China

[2] Hunan Provincial Key Laboratory of Vehicle Power and Transmission System, Hunan Institute of Engineering, 88 Fuxing East Road, Xiangtan 411104, China

[3] SJTU Paris Elite Institute of Technology, Shanghai Jiao Tong University, Shanghai 200240, China

[*]Corresponding author: wenmingz@sjtu.edu.cn

Content:

S1. Modeling and analysis of the magnetic field

S2. Calculation of magnetic susceptibility of paramagnetic solution

S3. Mechanical modeling of the dynamically rotating MagLev system

S4. Experimental setup and materials

Figure S1. Schematic of the “standard” MagLev configuration

Figure S2. Orientation of objects in three-dimensional space.

Figure S3. Three-dimensional spatial profiles of the magnetic field

Figure S4. Illustration of the geometric dimensions of the double ring-shaped magnets

Figure S5. Comparison of magnetic field between the theoretical calculation and fitted results

Figure S6. Schematic of the assembly process of the MagLev configuration

Figure S7. Prototype of the dynamically rotating MagLev

Figure S8. Calibration and density measurement of the 3D-printed materials

Figure S9. Schematic of the dimensions and body-fixed frame of references of the rods

Figure S10. Comparison of the static levitation of objects with different inclusion volumes

Figure S11. Comparison of the centroid position of the levitating rod (l_2=1 mm)

Table S1. Comparison of MagLev configuration for density-based measurement and characterization.

S1. Modeling and analysis of the magnetic field

The magnetic field generated by the ring-shaped magnet can be calculated based on the Coulombian model mentioned in the main text. The axially magnetized magnet ring is represented by two annular planes which are charged with a surface magnetic pole density $+\sigma^*$, $-\sigma^*$, respectively. Then the total magnetic field generated by the ring-shaped magnet is calculated by the application of the linear superposition principle, as given by Equation S1-S3,

$$H_r(r,z) = H_r^+(r,z) + H_r^-(r,z) \tag{S1}$$

$$H_z(r,z) = H_z^+(r,z) + H_z^-(r,z) \tag{S2}$$

$$H_\theta(r,z) = H_\theta^+(r,z) + H_\theta^-(r,z) = 0 \tag{S3}$$

where the azimuthal component H_θ (r, z) equals 0 due to the cylindrical symmetry. The three-dimensional components of the axially magnetized ring are expressed over complete elliptical integrals of the first and second kind that can be given by

$$H_r^+(r,z) = \frac{\sigma^*}{\pi\mu_0}\sum_{n=1}^{2}(-1)^{n-1}\frac{1}{k_n^+}\sqrt{\frac{r_n}{r}}\left[E(k_n^+) - (1-\frac{k_n^{+2}}{2})K(k_n^+)\right] \tag{S4}$$

$$H_r^-(r,z) = \frac{-\sigma^*}{\pi\mu_0}\sum_{n=1}^{2}(-1)^{n-1}\frac{1}{k_n^-}\sqrt{\frac{r_n}{r}}\left[E(k_n^-) - (1-\frac{k_n^{-2}}{2})K(k_n^-)\right] \tag{S5}$$

where the geometric parameters include: inner radius r_1, outer radius r_2 and height h. $\mu_0=4\pi\times10^{-7}$ $(\mathrm{N\cdot A^{-2}})$ represents the permeability of free space. $K(k)$ denotes the complete elliptic integral of the first kind, and $\mathrm{E}(k)$ denotes the complete elliptic integral of the second kind. k_n^+ and k_n^- can be calculated by

$$k_n^+ = \sqrt{4rr_n / ((r+r_n)^2 + (z-h)^2)} \tag{S6}$$

$$k_n^- = \sqrt{4rr_n / ((r+r_n)^2 + z^2)} \tag{S7}$$

The axial component H_z (r, z) can be given as

$$\begin{aligned} H_z^+(r,z) = {} & \frac{\sigma^*}{4\pi\mu_0(z-h)}\sum_{n=1}^{2}(-1)^{n-1}\frac{k_n^+}{\sqrt{rr_n}} \\ & \times\left[(D^+ - r)(D^+ - r_n)\Pi(h_1^+, k_n^+) + (D^+ + r)(D^+ + r_n)\Pi(h_2^+, k_n^+)\right] \end{aligned} \tag{S8}$$

$$H_z^-(r,z)=\frac{-\sigma^*}{4\pi\mu_0 z}\sum_{n=1}^{2}(-1)^{n-1}\frac{k_n^-}{\sqrt{rr_n}}\times\left[(D^- - r)(D^- - r_n)\Pi(h_1^-,k_n^-)+(D^- + r)(D^- + r_n)\Pi(h_2^-,k_n^-)\right] \quad (S9)$$

where Π(*n*, *k*) represents the complete elliptic integral of the third kind. D^+, D^-, h_n^+ and h_n^- can be expressed by

$$D^+=\sqrt{r^2+(z-h)^2} \quad (S10)$$

$$D^-=\sqrt{r^2+z^2} \quad (S11)$$

$$h_n^+=2r\Big/(r+(-1)^{n-1}\sqrt{r^2+(z-h)^2}) \quad (S12)$$

$$h_n^-=2r/(r+(-1)^{n-1}\sqrt{r^2+z^2}) \quad (S13)$$

Figure S4 shows the schematic of the geometric dimensions of the double ring-shaped magnets. The two magnets with the same dimension ($r_{out}\times r_{in}\times h$=50 mm×25 mm×15 mm, N40) are positioned apart by *d*=17 mm. The magnetic field can be obtained by superimposing the magnetic field of the two permanent magnet rings. As shown in Figure S4, the radical component $H_{Ar}(r, z)$ of the magnetic field generated by the magnet A can be given by

$$H_{Ar}^+(r,z)=\frac{\sigma^*}{\pi\mu_0}\sum_{n=1}^{2}(-1)^{n-1}\frac{1}{k_{An}^+}\sqrt{\frac{r_n}{r}}\left[E(k_{An}^+)-(1-\frac{k_{An}^{+\,2}}{2})K(k_{An}^+)\right] \quad (S14)$$

$$H_{Ar}^-(r,z)=\frac{-\sigma^*}{\pi\mu_0}\sum_{n=1}^{2}(-1)^{n-1}\frac{1}{k_{An}^-}\sqrt{\frac{r_n}{r}}\left[E(k_{An}^-)-(1-\frac{k_{An}^{-\,2}}{2})K(k_{An}^-)\right] \quad (S15)$$

where $k_{An}^+=\sqrt{4rr_n/((r+r_n)^2+(z+d/2)^2)}$, $k_{An}^-=\sqrt{4rr_n/((r+r_n)^2+(z+(d/2+h))^2)}$. Similarly, we can obtain the radical component $H_{Br}(r, z)$ of the magnetic field generated by the magnet B, which can be expressed by

$$H_{Br}^+(r,z)=\frac{\sigma^*}{\pi\mu_0}\sum_{n=1}^{2}(-1)^{n-1}\frac{1}{k_{Bn}^+}\sqrt{\frac{r_n}{r}}\left[E(k_{Bn}^+)-(1-\frac{k_{Bn}^{+\,2}}{2})K(k_{Bn}^+)\right] \quad (S16)$$

$$H_{Br}^-(r,z)=\frac{-\sigma^*}{\pi\mu_0}\sum_{n=1}^{2}(-1)^{n-1}\frac{1}{k_{Bn}^-}\sqrt{\frac{r_n}{r}}\left[E(k_{Bn}^-)-(1-\frac{k_{Bn}^{-\,2}}{2})K(k_{Bn}^-)\right] \quad (S17)$$

where $k_{Bn}^+=\sqrt{4rr_n/((r+r_n)^2+(z-d/2)^2)}$, $k_{Bn}^-=\sqrt{4rr_n/((r+r_n)^2+(z-(d/2+h))^2)}$. Then the magnetic induction intensity components B_x, B_y generated by the double magnet rings at any point *M* (*x*, *y*) in the *x*-*y* plane can be expressed as

$$B_x = \mu_0 (H_{\mathrm{A}r} + H_{\mathrm{B}r}) \cdot \frac{x}{\sqrt{x^2 + y^2}} \tag{S18}$$

$$B_y = \mu_0 (H_{\mathrm{A}r} + H_{\mathrm{B}r}) \cdot \frac{y}{\sqrt{x^2 + y^2}} \tag{S19}$$

Figure S3 plots the three-dimensional spatial profiles of the magnetic field in the *x*-*y* plane, *y*-*z* plane and *x*-*z* plane. It can be seen that the magnetic flux density in the *x*-*y* plane is smaller than that in the *y*-*z* plane and *x*-*z* plane. This distribution feature of this magnetic field controls the alignment of the object in the *x*-*y* plane. Figure 2 in the main text demonstrates that the vector products of the magnetic field intensity exhibit obvious nonlinearity. To better analysis the effect of the nonlinear magnetic field on the levitation, a cubic polynomial is used to approximate the magnetic field components B_x, B_y in the *x*-*y* plane, which can be given by

$$B_x = k_1 x + k_2 x^3 + k_3 x y^2 \tag{S20}$$

$$B_y = k_4 y + k_5 y^3 + k_6 x^2 y \tag{S21}$$

where k_n (n=1, …, 6) represent the fitting coefficients obtained by Curve Fitting in MATLAB R2021b. Figure S5 shows the comparison of the theoretical calculated magnetic field component B_x and fitted magnetic field B_{xf}. As shown in Figure S5a, the cubic polynomial can better describe the nonlinear relationship between the curved surface of the magnetic field and the spatial position (R^2>0.99). Figure S5b plots the difference of the magnetic field between the theoretical calculation and fitting results.

S2. Calculation of magnetic susceptibility of paramagnetic solution

The magnetic susceptibility of the paramagnetic solution depends on the temperature and concentration of the solution. We calculated χ_s using Equation (S22) as described by Mirica et al.,

$$\chi_{\mathrm{s}} = \chi_{\mathrm{p}} c - 9 \times 10^{-6} \tag{S22}$$

where χ_p represents the molar magnetic susceptibility of the paramagnetic salt, *c* is the concentration of the solution, and -9×10^{-6} is the magnetic susceptibility of water. In this work, we use $MnCl_2$ aqueous solution as the paramagnetic solution. The molar magnetic

susceptibility of $MnCl_2$ for room temperature T=296 K is $\chi_{\mathrm{p}} = 1.858\times10^{-4}$. For the diamagnetic materials mentioned in this work, $\chi_{\mathrm{o}} = -5\times10^{-5}$ is estimated as the diamagnetic susceptibility. For the diamagnetic materials in the paramagnetic solution, it can be seen that $\chi_{\mathrm{s}} \gg \chi_{\mathrm{o}}$. Thus, the diamagnetic objects are considered to be isotropic in terms of magnetics.

S3. Mechanical modeling of the dynamically rotating MagLev system

To better describe the dynamic behavior of the levitating object in the dynamically rotating MagLev system, we establish a mechanical model to investigate the effect of the nonlinear magnetic field on the levitation equilibrium as well as to reveal the mechanism of the dynamic centrifugal force on the levitation stability, and predict the dynamic response of different inhomogeneous objects.

In the main text, we define two coordinates: the MagLev frame of reference, the *x*-, *y*- and *z*- axes, with the origin placed at the center of the middle plane of these two magnets, and the chosen body-fixed, principal frame of reference, the *u*-, *v*- and *w*-axes, with the origin placed at the centroid of the levitating object. It's shown that the MagLev configuration controls the alignment of the levitating object in the *x*-*y* plane. Hence, the motion of the levitating object in the magnetic field can be described by the coordinate (x, y, ψ) of position and angle. Any manipulation of the levitating object in the MagLev can be treated as a pure rotation by $R_z(\psi)$ plus an in-plane translation, which can be given by

$$\begin{bmatrix} x \\ y \\ z \end{bmatrix} = R_z(\psi)\begin{bmatrix} u \\ v \\ w \end{bmatrix} + \begin{bmatrix} x_0 \\ y_0 \\ z_0 \end{bmatrix} \tag{S23}$$

where (x_0, y_0, z_0) is the spatial position of the origin of the body-fixed frame of reference, and $R_z(\psi)$ denotes the rotation matrix,

$$R_z(\psi) = \begin{bmatrix} \cos\psi & -\sin\psi & 0 \\ \sin\psi & \cos\psi & 0 \\ 0 & 0 & 1 \end{bmatrix} \tag{S24}$$

We consider that an inhomogeneous object, of volume *V*, levitates in a paramagnetic medium in the MagLev device. A nonconvex potential energy landscape is responsible for the existence of stable equilibrium states (including the levitation position and angular

orientation). We decompose the effective potential energy into the magnetic contribution, gravitational contribution and centrifugal potential energy. The object has an inhomogeneous magnetic susceptibility $\chi_o(u, v, w)$ and density $\rho_o(u, v, w)$. Thereinto, we neglect the anisotropic effect of the magnetic susceptibility of the object as $\chi_o \ll \chi_s$, and assume $\triangle\chi$ is homogeneous throughout the object. The potential energy due to the magnetic field within the volume of the arbitrary object can be described as

$$U_{\text{mag}} = -\int_V \frac{(\chi_o - \chi_s)}{2\mu_0} \boldsymbol{B} \cdot \boldsymbol{B}\, dV = -\int_V \frac{(\chi_o - \chi_s)}{2\mu_0} (B_x^2 + B_y^2)\, dV \qquad \text{(S25)}$$

where B_x, B_y can be calculated by the cubic polynomial (as shown in Equations S20-S21). For the inhomogeneous object, the heterogeneity in density, i.e., the distribution of density within the volume, can be described as a function. The potential energy due to the gravitational field within the volume of the object can be given by

$$U_{\text{grav}} = \int_V \triangle\rho \boldsymbol{g} \cdot \boldsymbol{y}\, dV \qquad \text{(S26)}$$

where $\triangle\rho = \rho_o - \rho_s$ represents the density of the object relative to the paramagnetic solution. $\boldsymbol{g}$ is gravitational acceleration. For the object levitated in the MagLev device in the non-inertial rotating system, the centrifugal potential energy within the volume of the object can be expressed as

$$U_c = -\int_V \left(\frac{1}{2}\triangle\rho\omega^2 (D + x)^2\right) dV \qquad \text{(S27)}$$

where ω is the rotating speed of the system, D is the eccentric distance. In Equations S25-S27, dV is the volume element, and the spatial location position can be obtained as shown in Equation S23.

The effective potential energy of the levitating object is $U = U_{\text{mag}} + U_{\text{grav}} + U_c$. Stable equilibrium occurs where the local minimum of the total energy U exists. Finding the equilibrium levitation involves minimizing simultaneously the energy associated with the levitation position as well as orientation, which can be given by

$$\nabla U = 0 \quad \Leftrightarrow \quad \begin{aligned} \frac{\partial U}{\partial x_0} = \frac{\partial U_{\mathrm{mag}}}{\partial x_0} + \frac{\partial U_{\mathrm{grav}}}{\partial x_0} + \frac{\partial U_{\mathrm{c}}}{\partial x_0} = 0 \\ \frac{\partial U}{\partial y_0} = \frac{\partial U_{\mathrm{mag}}}{\partial y_0} + \frac{\partial U_{\mathrm{grav}}}{\partial y_0} + \frac{\partial U_{\mathrm{c}}}{\partial y_0} = 0 \\ \frac{\partial U}{\partial \psi} = \frac{\partial U_{\mathrm{mag}}}{\partial \psi} + \frac{\partial U_{\mathrm{grav}}}{\partial \psi} + \frac{\partial U_{\mathrm{c}}}{\partial \psi} = 0 \end{aligned} \tag{S28}$$

Positions that energy extra occur can be obtained by numerically solving Equation S28. For the stable levitation, the positive curvature in the energy surface in every direction is necessary, that is,

$$\nabla \cdot \boldsymbol{F} = -\nabla^2 U < 0 \tag{S29}$$

Then we can find the stable levitation positions and orientations where the energy minima occur.

In order to avoid the influence of the geometry of levitating object, we consider the simple case of the cylindrical rod. The origin of the body-fixed principal frame of reference is positioned at the centroid (geometric center) of the object, and the *u*-axis is defined as the direction parallel to the length. Figure S9a and b show the schematic of the dimensions and body-fixed frame of references of the rods with local heterogeneity. The rod, with a diameter d_1, length l_1, is with a density of ρ_1, and the local heterogeneity characterized by an interior cylindrical inclusion with a diameter d_2, length l_2 and displacement t along the *u*-axis, is with a density of ρ_2. In this case, the magnetic potential energy can be calculated as

$$U_{\mathrm{mag}} = -\int_{-\frac{l_1}{2}}^{\frac{l_1}{2}} \int_0^{2\pi} \int_0^{\frac{d_1}{2}} \frac{(\chi_{\mathrm{o}} - \chi_{\mathrm{s}})}{2\mu_0} (B_x^2 + B_y^2) \cdot R dR d\theta du \tag{S30}$$

where we assume that the differences in χ_{o} over the volume of the object have a negligible effect. Based on Equation S26, we expand the gravitational potential energy into two terms, which can be given by

$$\begin{aligned} U_{\mathrm{grav}} = & \int_{-\frac{l_1}{2}}^{\frac{l_1}{2}} \int_0^{2\pi} \int_0^{\frac{d_1}{2}} (\rho_1 - \rho_{\mathrm{s}}) g \cdot (u \sin\psi + v\cos\psi + y_0) \cdot R dR d\theta du + \\ & \int_{-\frac{l_1}{2}+t-\frac{l_2}{2}}^{-\frac{l_1}{2}+t+\frac{l_2}{2}} \int_0^{2\pi} \int_0^{\frac{d_2}{2}} (\rho_2 - \rho_1) g \cdot (u \sin\psi + v\cos\psi + y_0) \cdot R dR d\theta du \end{aligned} \tag{S31}$$

where ρ_{s} is the density of the paramagnetic solution, t denotes the distance from the center of the inclusion to the end face. Similarly, we can obtain the centrifugal potential energy that can be given by

$$U_{\mathrm{c}}=\int_{-\frac{l_1}{2}}^{\frac{l_1}{2}}\int_0^{2\pi}\int_0^{\frac{d_1}{2}}(-\frac{1}{2}(\rho_1-\rho_{\mathrm{s}})\omega^2(D+u\cos\psi-v\sin\psi+x_0)^2)\cdot RdRd\theta du$$
$$+\int_{-\frac{l_1}{2}+t-\frac{l_2}{2}}^{-\frac{l_1}{2}+t+\frac{l_2}{2}}\int_0^{2\pi}\int_0^{\frac{d_2}{2}}(-\frac{1}{2}(\rho_2-\rho_1)\omega^2(D+u\cos\psi-v\sin\psi+x_0)^2)\cdot RdRd\theta du \quad \text{(S32)}$$

where ω denotes the rotating speed, D represents the eccentric distance of the MagLev device. The centrifugal potential energy U_{c} is tunable for the varying rotating speed and eccentric distance. By submitting Equations S30-S32 to Equations S28-S29, the equilibrium levitation states can be calculated and predicted.

Gradients in density are commonly found in natural and artificial materials. As another demonstration, here we use the dynamically rotating MagLev to characterize the gradient density which cannot be detected in the standard MagLev. Similarly, we construct cylindrical models with gradients in density (as shown in Figure S9 c and d), and change the density gradients by varying the diameter of the conical end face. For an object with gradient in density along the axis of symmetry, the center of mass and centroid are, in general, located at the same point. In this case, the gravitational potential energy U_{grav} can be given as

$$U_{\mathrm{grav}}=\int_{-\frac{l_1}{2}}^{\frac{l_1}{2}}\int_0^{2\pi}\int_0^{\frac{d_1}{2}}(\rho_1-\rho_{\mathrm{s}})g\cdot(u\sin\psi+v\cos\psi+y_0)\cdot RdRd\theta du$$
$$+\int_0^{\frac{m_2}{2}}\int_0^{2\pi}\int_{\frac{2l_1}{m_2}R-\frac{l_1}{2}}^{\frac{l_1}{2}}(\rho_2-\rho_1)g\cdot(u\sin\psi+v\cos\psi+y_0)\cdot Rdud\theta dR \quad \text{(S33)}$$

where m_2 represents the diameter of the conical end face and determines the gradient in density. In the uniformly rotating non-inertial reference, the centrifugal potential energy can be given by

$$U_{\mathrm{c}}=\int_{-\frac{l_1}{2}}^{\frac{l_1}{2}}\int_0^{2\pi}\int_0^{\frac{d_1}{2}}(-\frac{1}{2}(\rho_1-\rho_{\mathrm{s}})\omega^2(D+x)^2)\cdot RdRd\theta du$$
$$+\int_0^{\frac{m_2}{2}}\int_0^{2\pi}\int_{\frac{2l_1}{m_2}R-\frac{l_1}{2}}^{\frac{l_1}{2}}(-\frac{1}{2}(\rho_2-\rho_1)\omega^2(D+x)^2)\cdot Rdud\theta dR \quad \text{(S34)}$$

Then the effective potential energy of the levitating object can be obtained by $U=U_{\mathrm{mag}}+U_{\mathrm{grav}}+U_{\mathrm{c}}$ .The energy minimum value can be calculated after submitting Equations S30, S33 and S34 to Equations S28-S29.

S4. Experimental setup and materials

In this work, a pair of ring-shaped magnets (N40, Jiuci, Beijing, China) are made of NdFeB, and the surfaces of the magnets are coated with nickel-copper-nickel. The dimension of the magnet ring is $r_{out} \times r_{in} \times h$=50 mm×25 mm×15 mm. The assembly of these two magnets may be very dangerous due to the size and the arrangement. The two magnets, with like poles facing each other, produce repulsive force. The smaller the distance between the magnets is, the larger the magnetic repulsive force is. Hence, checking the polarities of the magnets with a small, weak magnet before the assembly is suggested.

Here we use a pair of guide rails to assist in the assembling process of the two magnets. Figure S6 shows the assembling process of the MagLev configuration. A set of custom aluminum holders is designed to secure the magnets mechanically and fixed on the rotating disc. First, a pair of holders with the semicircular shape is designed and fabricated to mount the magnet ring. One of the holders, with an L-shaped structure, can be fixedly connected to the guide rail and the rotating disc. By rotating the handwheel of the guide rail, the distance between the two magnet rings is adjusted. A pair of п-shaped holders is designed and used to mount these two magnets along the thickness direction, thus ensuring the constant distance between the two magnets. Finally, the whole MagLev configuration can be mounted on the rotating disc.

To enable stable and reliable rotation, we use a direct drive rotary motor (ADR175-A102, Akribis, China) and an AC motor controller (MR-J4, Akribis, China) to modulate the output rotary motion. An aluminum disc ($d \times h = 485\ \text{mm} \times 15\ \text{mm}$) is designed and installed on the output shaft end of the rotary motor. The aluminum disc surface is fabricated with a series of threaded holes so that the eccentric distance of the MagLev configuration could be adjusted. The prototype of the dynamically rotating MagLev is shown in Figure S7. A transparent container filled with the paramagnetic solution is fixed between the magnets. In the main text, we use $MnCl_2$ (Macklin Inc.) aqueous solution as the paramagnetic medium. The experimental images are collected in real time by a mobile phone (iPhone 6, Apple Inc.), which is mounted on a rotating disc by a custom-made fixture. The density standard beads

used here are purchased from American Density Materials, Inc. The additively manufacturing inhomogeneous rods are fabricated by a multi-material 3D printer (J750, Stratasys, United States). For the inhomogeneous objects, the cylindrical rods are made out of white resin material (Verowhite, Stratasys) that incorporates a series of inclusions out of transparent soft rubber material (Agilus 30, Stratasys). The densities of these two materials are calibrated and measured as shown in Figure S8.

Figure S1. Schematic of the "standard" MagLev configuration.

The "standard" MagLev configuration consists of two antialigned magnets (parallel with like-poles facing each other) and a sample container filled with paramagnetic solution sandwiched between these magnets, forming a potential well at the midpoint between these two magnets. Objects can be stably levitated when the buoyancy-corrected gravitational force ($\boldsymbol{F}_g$) balances the magnetic force ($\boldsymbol{F}_{mag}$), and the shape as well as heterogeneity in density determines the levitation orientation.

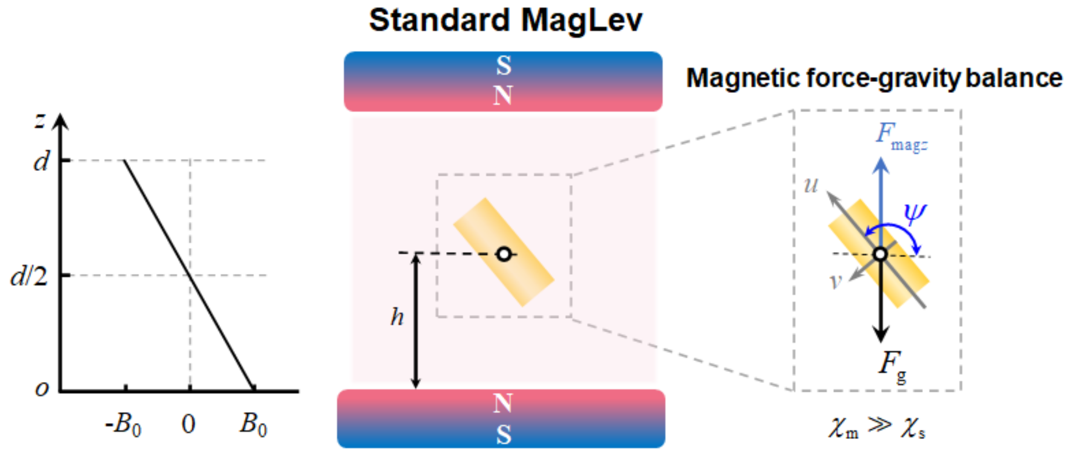

Figure S2. Orientation of objects in three-dimensional space.

(a) Orientation in the *x*-*y* plane. (b) Orientation out of *x*-*y* plane.

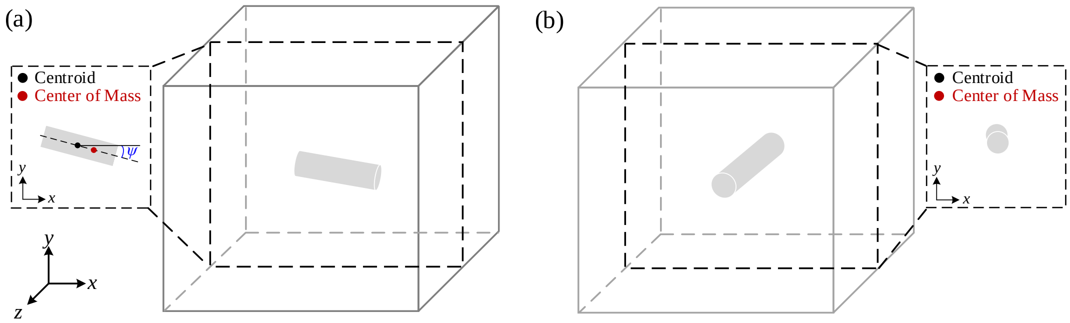

Figure S3. Three-dimensional spatial profiles of the magnetic field.

The dimension of the two ring-shaped magnets is r_{out}×r_{in}×h=50 mm×25 mm×15 mm. The finite element analysis of the magnetic field is carried out based on COMSOL Multiphysics. Through the AC/DC module, the static magnetic field is simulated and the ditribution of the magnetic field is plotted. A slice of the three-dimensional magnetic field (a) in the *x*-*y* plane with respect to the *z*-axis, (b) in the *y*-*z* plane with respect to the *x*-axis, and (c) in the *x*-*z* plane with respect to the *y*-axis.

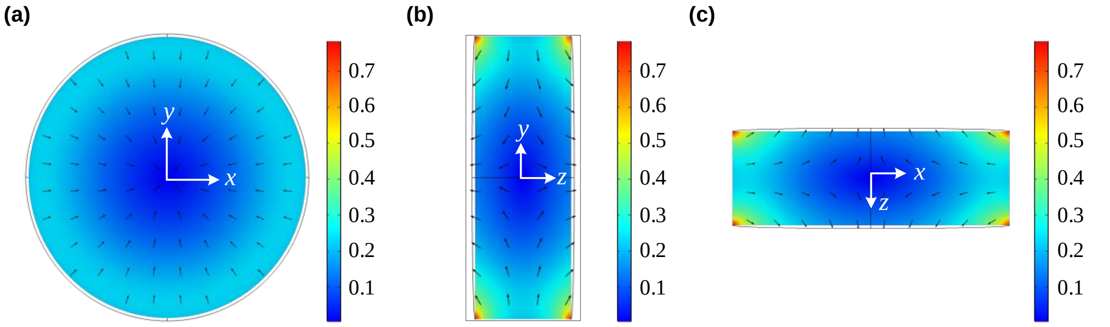

Figure S4. Illustration of the geometric dimensions of the double ring-shaped magnets. A pair of ring-shaped magnets (r_{out}×r_{in}×h=50 mm×25 mm×15 mm, N40) are positioned apart by d=17 mm, with like-poles facing each other. We assume that the inner surfaces are charged with a surface magnetic pole density +σ* and the outer ones are charged with the opposite surface magnetic pole density −σ*.

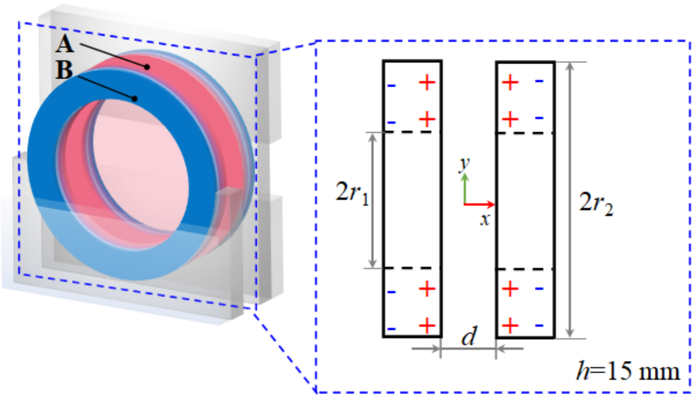

Figure S5. Comparison of magnetic field between the theoretical calculation and fitted results.

We use a cubic polynomial to approximate the magnetic field components B_x, B_y in the x-y plane. The coefficients are obtained by Curve Fitting in MATLAB R2021b. (a) Comparison of the theoretical calculated magnetic field component B_x and fitted magnetic field B_{xf}. (b) Distribution of the difference between the theoretical calculated magnetic field component B_x and fitted magnetic field B_{xf}.

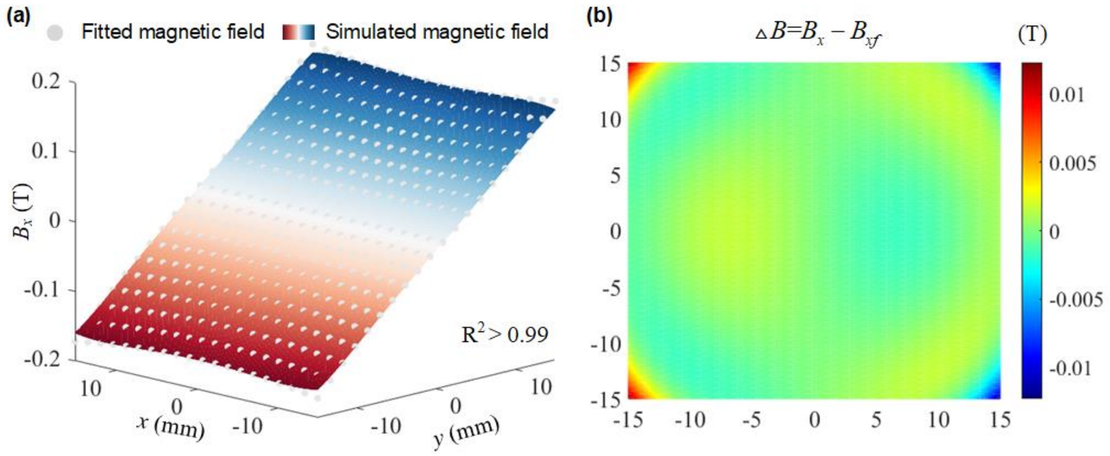

Figure S6. Schematic of the assembly process of the MagLev configuration.

(a) The two magnets with like poles facing each other are assembled with the aid of two guide rails. A pair of aluminum holders with the semicircular shape is designed and fabricated to mount the magnet ring. One of the holders, with an L-shaped structure, can be fixedly connected to the guide rail and the rotating disc. By rotating the handwheel of the guide rail, the distance between the two magnet rings is adjusted. Finally, a pair of ⊓-shaped holders is designed and used to mount these two magnets along the thickness direction, thus ensuring the constant distance between the two magnets. (b) Schematic of the assembled MagLev configuration.

(a)

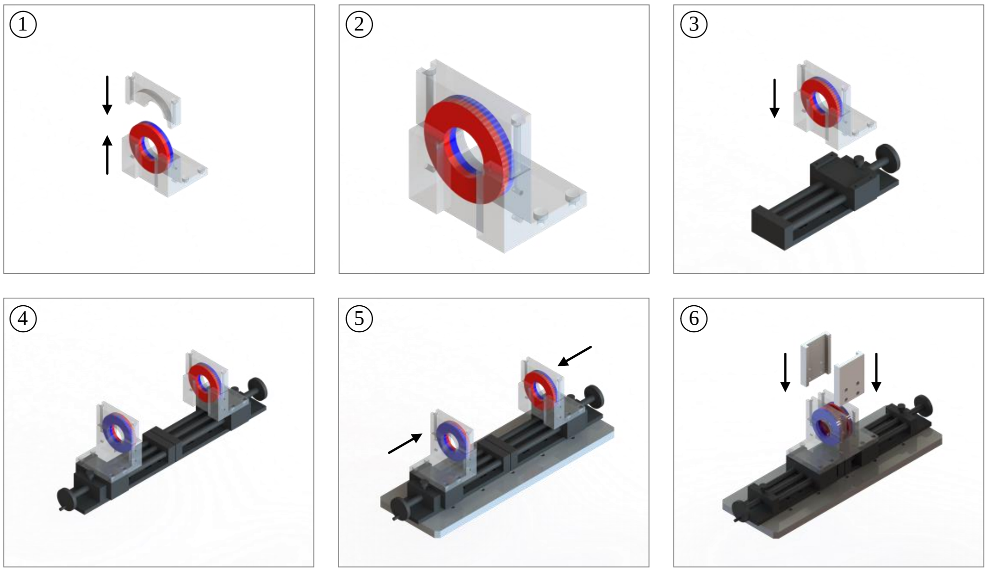

(b)

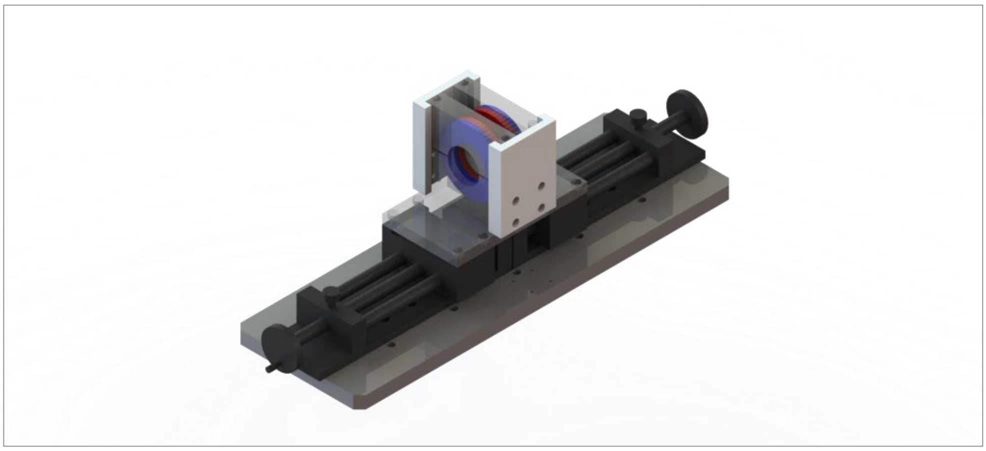

Figure S7. Prototype of the dynamically rotating MagLev.

A direct drive rotary motor (ADR175-A102, Akribis, China) and an AC motor controller (MR-J4, Akribis, China) to modulate the output rotary motion are employed in the system. An aluminum disc ($d \times h = 485\ \mathrm{mm} \times 15\ \mathrm{mm}$) with threaded holes is installed on the output shaft end of the rotary motor. The aluminum disc surface is fabricated with a series of threaded holes so that the eccentric distance of the MagLev configuration could be adjusted. The MagLev configuration is eccentrically fixed on the rotating disc, and a transparent container filled with the paramagnetic solution is fixed between the magnets. The experimental images are collected in real time by a mobile phone (iPhone 6, Apple Inc.), which is mounted on a rotating disc by a custom-made fixture.

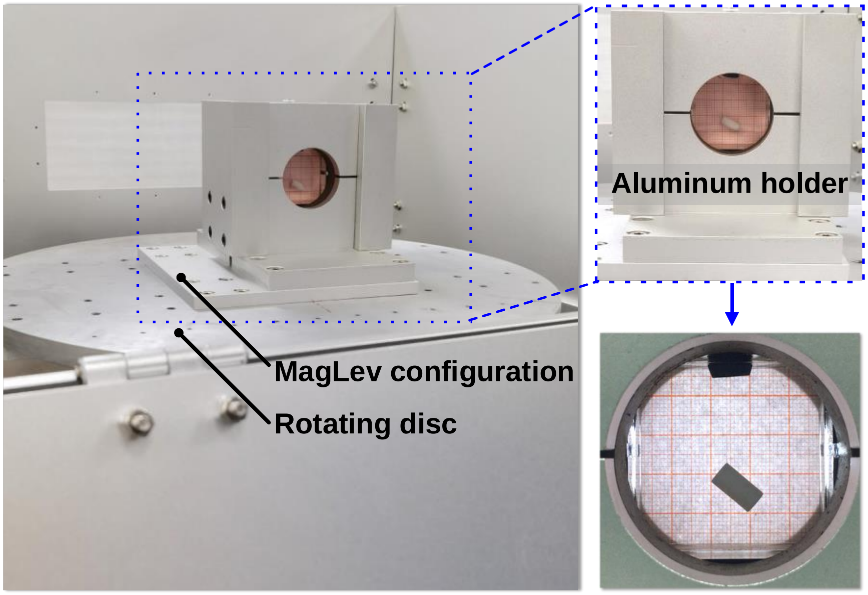

Figure S8. Calibration and density measurement of the 3D-printed materials.

The density of two 3D-printeing materials, i.e., white resin (Verowhite, Stratasys) and soft rubber (Agilus 30, Stratasys), is measured by the dynamically rotating MagLev. Two spheres with a diameter of 4 mm and a yellow standard density ball with a density of 1.170±0.0002 g/cm^3 are levitated in an aqueous solution of 1.78 M $MnCl_2$ solution. The eccentric distance is set D=26 mm, and the rotational speed is continuously increased from ω=50 r/min to ω=150 r/min in increments of 10 r/min. The density of the white resin is ρ_1=1.203 g/cm^3 and the density of the soft rubber is ρ_2=1.146 g/cm^3 by repeating the experiment five times.

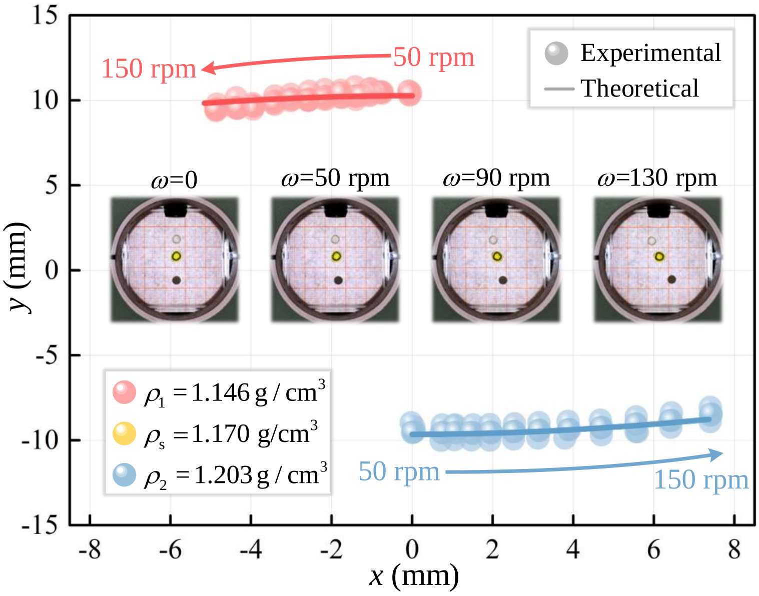

Figure S9. Schematic of the dimensions and body-fixed frame of references of the rods. We use the multi-material 3D printer (Stratasys, J750) capable of printing materials of dissimilar densities to construct the local heterogeneity in density and gradient in density. (a) The local heterogeneity in density is constructed by incorporating a series of cylindrical inclusions. (b) The gradient in density is constructed by incorporating a series of conical inclusions.

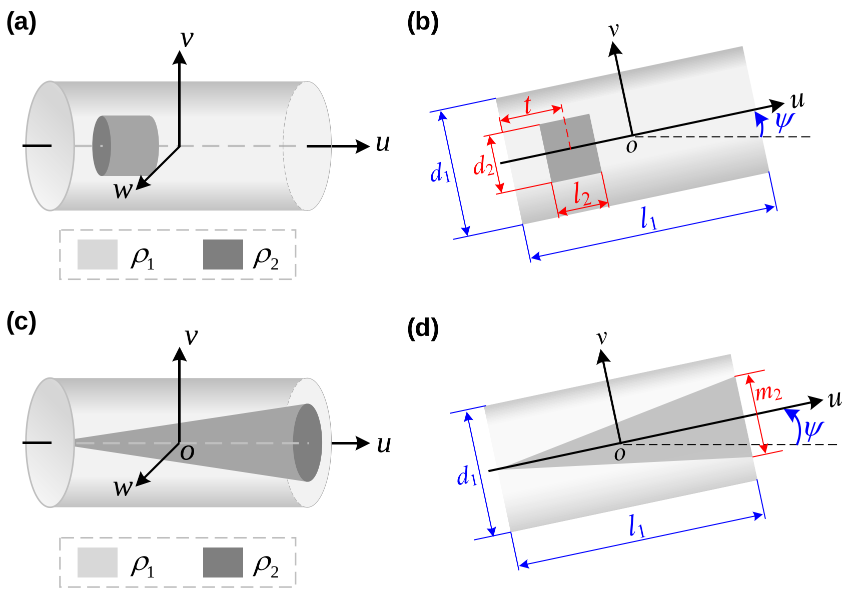

Figure S10. Comparison of the static levitation of objects with different inclusion volumes

We focus on three kinds of rods (l_1=12 mm, d_1= 6 mm) containing cylindrical inclusions characterized by different heights (l_2=1 mm, 2 mm and 3 mm), but with the same diameter of d_2=3 mm and same inclusion distance (t=2 mm) to the end face. The rods are levitated in 1.78 M $MnCl_2$ in the MagLev. (a) Theoretical results and experimental data of the statically levitated rods with different inclusions. (b) Images of the levitation of rods with different inclusions.

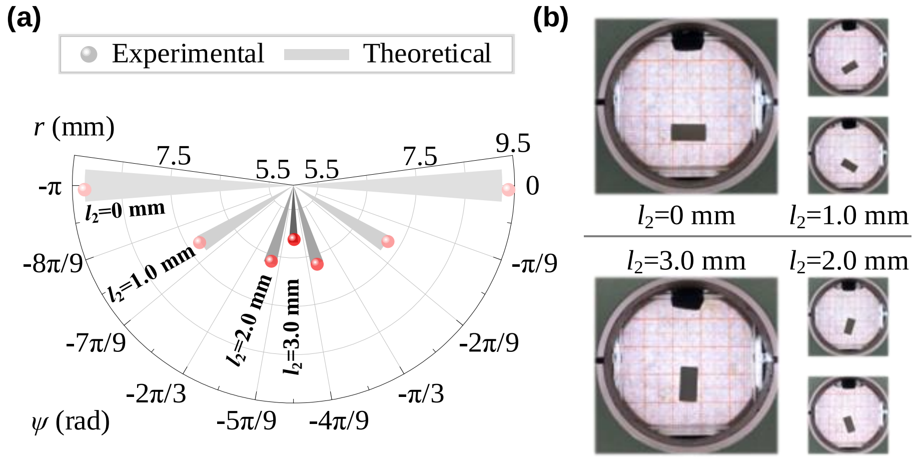

Figure S11. Comparison of the centroid position of the levitating rod (l_2=1 mm).

The centroid positions in State I and II are close but not the same since the local heterogeneity makes the centroid not coincide with the center-of-mass.

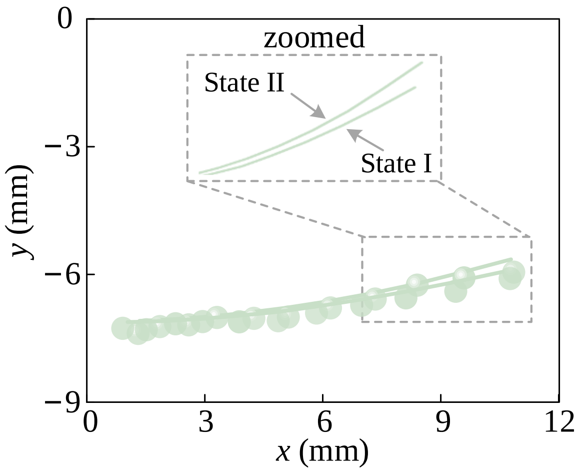

Table S1. Comparison of MagLev configuration for density-based measurement and characterization.

Homogeneous Density Measurement									Heterogeneous Characterization			
Type	Standard[1]	Tilted[2]	High-sensitivity[3]	High-throughput[4]	Single-ring[5]	Axial[6]	Axial-circular[7]	Electro-magnet[8]	Standard[1]	Electro-magnet[8]	This work	Type
Sample	Liquid, solid	Gas, liquid, solid	Liquid, solid	Gas, liquid, solid	Liquid, solid	Gas, liquid, solid	Liquid, solid	Solid	Solid	Solid	Solid	Sample
Solution	Paramagnetic salt solution	Paramagnetic salt solution, aqueous dextran	Co-solutes, including paramagnetic and simple salts	Paramagnetic salt solution, paramagnetic chelates	Paramagnetic salt solution	Paramagnetic salt solution	Paramagnetic salt solution, paramagnetic chelates	Paramagnetic salt solution	Co-solutes, including paramagnetic and simple salts	Paramagnetic salt solution	Paramagnetic salt solution	Solution
Measurement range (g/cm^3)	0.8~3	0~23	0.8~3	0~9	-	0~3.7	0.8~3	0.8~2.308	√	√	√	Asymmetric defect
Measurement resolution (g/cm^3)	10^{-2}~10^{-4}	1~10^{-2}	10^{-2}~10^{-6}	10^{-1}~10^{-4}	-	-	10^{-2}~10^{-6}	-	×	×	√	Symmetric defect
Operational simplicity	High	Medium	High	High	High	High	High	High	High	Medium	High	Operational simplicity
Cost	Low	Medium	Low	Medium	Low	Low	Low	High	Low	High	Medium	Cost
Energy Input	No	No	No	No	No	No	No	Yes	No	Yes	Yes	Energy Input

References:

1. Mirica, K. A.; Shevkoplyas, S. S.; Phillips, S. T.; Gupta, M.; Whitesides, G. M., Measuring Densities of Solids and Liquids Using Magnetic Levitation: Fundamentals. Journal of the American Chemical Society 2009, 131 (29), 10049-10058.
2. Nemiroski, A.; Soh, S.; Kwok, S. W.; Yu, H. D.; Whitesides, G. M., Tilted Magnetic Levitation Enables Measurement of the Complete Range of Densities of Materials with Low Magnetic Permeability. Journal of the American Chemical Society 2016, 138 (4), 1252-1257.
3. Nemiroski, A.; Kumar, A. A.; Soh, S.; Harburg, D. V.; Yu, H. D.; Whitesides, G. M., High-Sensitivity Measurement of Density by Magnetic Levitation. Analytical Chemistry 2016, 88 (5), 2666-2674.
4. Ge, S.; Wang, Y.; Deshler, N. J.; Preston, D. J.; Whitesides, G. M., High-Throughput Density Measurement Using Magnetic Levitation. Journal of the American Chemical Society 2018, 140 (24), 7510-7518.

5. Zhang, C.; Zhao, P.; Gu, F.; Xie, J.; Xia, N.; He, Y.; Fu, J., Single Ring Magnetic Levitation Configuration for Object Manipulation and Density-based Measurement. Analytical Chemistry 2018, 90 (15), 9226-9233.
6. Ge, S.; Whitesides, G. M., “Axial” Magnetic Levitation Using Ring Magnets Enables Simple Density-Based Analysis, Separation, and Manipulation. Analytical Chemistry 2018, 90 (20), 12239-12245.
7. Zhang, C.; Zhao, P.; Gu, F.; Zhang, X.; Xie, J.; He, Y.; Zhou, H.; Fu, J.; Turng, L.-S., Axial-Circular Magnetic Levitation: A Three-Dimensional Density Measurement and Manipulation Approach. Analytical Chemistry 2020, 92 (10), 6925-6931.
8. Jia, Y.; Zhao, P.; Xie, J.; Zhang, X.; Zhou, H.; Fu, J., Single-electromagnet levitation for density measurement and defect detection. Frontiers of Mechanical Engineering 2021, 16 (1), 186-195.
